# Tailoring the Ross procedure for patients with aortic regurgitation

**DOI:** 10.1016/j.xjtc.2021.06.008

**Published:** 2021-06-08

**Authors:** Amine Mazine, Ismail El-Hamamsy

**Affiliations:** a Division of Cardiac Surgery, Department of Surgery, University of Toronto, Toronto, Ontario, Canada; b Department of Cardiovascular Surgery, Mount Sinai Hospital, Icahn School of Medicine at Mount Sinai, New York, NY

**Keywords:** Ross procedure, aortic regurgitation, pulmonary autograft

**Figure:**
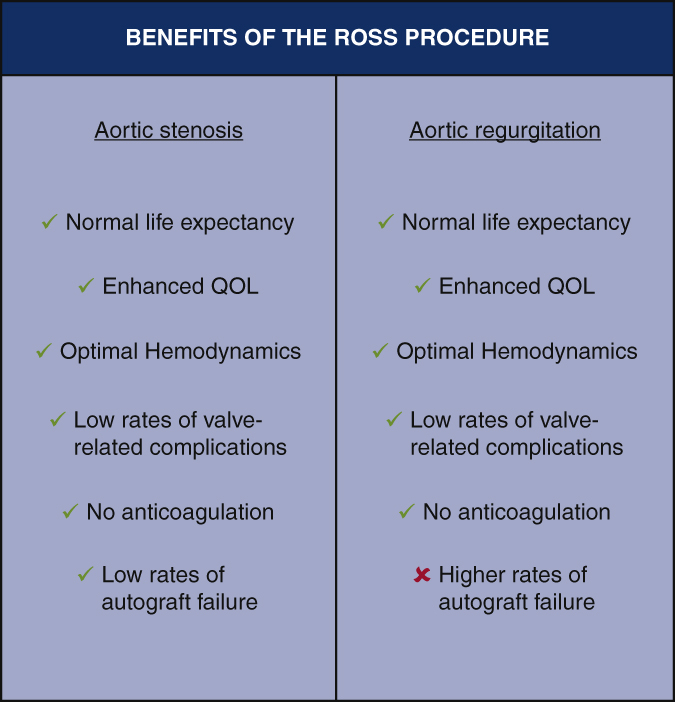
Benefits of the Ross procedure in patients with aortic stenosis and aortic regurgitation.

**Central Message**

With proper technical refinements and strict postoperative blood pressure control, the Ross procedure is an excellent operation for treating nonrepairable aortic regurgitation in young adults.

See Commentaries on pages 390, 392, and 394.

Aortic valve repair and aortic valve-sparing operations represent the best options to treat aortic regurgitation (AR) in nonelderly adults.^1^^,^^2^ When performed by expert surgeons, these operations are safe and result in good durability and freedom from valve-related complications, leading to excellent long-term survival.^3^^,^^4^ When the aortic valve cannot be repaired or spared, valve replacement becomes essential. The vast majority of patients who undergo aortic valve replacement (AVR) will receive a bioprosthetic or mechanical valve. Numerous large studies have demonstrated that nonelderly adults who receive prosthetic valves—be they biological or mechanical—have a survival disadvantage compared with the age- and sex-matched general population.^5^^,^^6^ Importantly, the observed excess mortality is inversely proportional to patient age at the time of surgery (ie, the youngest patients have the largest excess mortality), owing to the higher functional demand and longer exposure to potential valve-related complications in young adults with prosthetic valves.^7^, ^8^, ^9^

In recent years, increasing recognition of the suboptimal outcomes of prosthetic AVR in young and middle-aged adults has led to a resurgence of interest in the Ross procedure.^10^ First described by Donald Ross in 1967,^11^ the Ross procedure is the only replacement operation that allows for long-term viability of the aortic root. Owing to its unique biological and hemodynamic properties, the Ross procedure is an attractive option for nonelderly adults undergoing AVR. In expert centers, it carries a similar operative risk as conventional AVR^12^ and is associated with low rates of valve-related complications, excellent quality of life, and long-term survival equivalent to that of the age- and sex-matched general population.^13^ In addition, several recent publications have demonstrated that in appropriately selected patients, the Ross procedure provides superior outcomes compared with prosthetic AVR.^14^, ^15^, ^16^, ^17^ As a result of this growing body of evidence, many experts now view the Ross procedure as the best operation to treat aortic stenosis (AS) in young and middle-aged adults.^18^^,^^19^ However, its use in patients presenting with AR remains a matter of debate. This is due to a higher risk of pulmonary autograft dilatation—and subsequent need for reintervention—in these patients. Indeed, several studies have consistently indicated an association between the presence of preoperative AR—particularly in the setting of a dilated aortic annulus—and an increased risk of reintervention.^20^, ^21^, ^22^, ^23^, ^24^, ^25^, ^26^, ^27^ As a result, many advocate against using the Ross procedure in the setting of AR. For instance, the 2013 Society of Thoracic Surgeons guidelines allocate a class III recommendation for the Ross procedure in patients with bicuspid aortic valve (BAV) and AR.^28^ Nevertheless, a careful examination of the data suggests otherwise. On the one hand, despite a higher reintervention hazard, the late survival benefit observed with the Ross procedure in AS is preserved in patients with AR. On the other hand, it has become apparent that the risk of reintervention can be mitigated by adapting the surgical technique and adjusting postoperative blood pressure management. Herein we review the contemporary evidence surrounding the use of the Ross procedure in patients with AR and describe the technical and medical modifications that make the Ross procedure the best operation to treat young adults with nonrepairable AR.

### Impact of Preoperative AR on Autograft Durability

Compared with patients who undergo surgery for AS, those who undergo surgery for AR tend to present at a younger age and have higher rates of congenital aortic valve anomalies (ie, bicuspid, unicuspid, or quadricuspid aortic valves), dilated aortic annuli, ascending aortic aneurysm, and size mismatch between the pulmonary and aortic roots. As a result of these factors, various clinical studies have demonstrated that patients undergoing the Ross procedure for AR are at greater risk of autograft dilatation and reoperation than those with AS. These studies are summarized in Table 1. Although a detailed review of these individual reports falls beyond the scope of this article, it should be noted that in most of these studies, the root replacement technique was heavily favored, and in most cases, no effective systematic root stabilization strategies or blood pressure control protocols were in place for patients with AR. Furthermore, none of the studies showed different long-term survival rates between the AS and AR groups. In other terms, the main benefit of the Ross procedure—preserved survival, which is predicated on the unique biologic and hemodynamic properties of the living pulmonary autograft—is preserved. Given this, rather than abandoning the Ross procedure, the question becomes “how can we improve durability in patients with AR”?

**Table 1:** Summary of studies comparing outcomes of the Ross procedure in adults with AS versus AR

Study	Patients, n	Mean age, y	BAV/UAV/QAV, %∗	AS/AR mixed AS-AR, %	Surgical technique	Annuloplasty, %	Annuloplasty type	Mean follow-up, y	Freedom from autograft reoperation
David et al. (2010)^24^	212	34 ± 9	82	50/36/13	RR (51%), SC/inclusion (49%)	46	Subcommissural plication and partial Dacron strip	10.1 ± 4.2	At 15 y: AS, 97% AR, 84%
Weimar et al. (2014)^27^	645	42 ± 14	58	32/29/33	RR (98%), SC (2%)	63	Dacron strip	8.4 ± 4.6	At 10 y: AS, 97% AR, 90%
Skillington et al. (2015)^29^	322	40 (range 15-63)	95	46/32/22	Inclusion (100%)	62	Circumferential ring (5%), partial ring (30%), partial ring and annular plication (25%), annular plication (2%)	9.8	At 18 y: 96% overall AS, n = 1 AR, n = 9 AS/AR, n = 1
Mastrobuoni et al. (2016)^30^	306	42 ± 10	59	68/31/0	SC (2%), RR (55%), inclusion (43%)	N/A	N/A	10.6	At 16 y: AS, 83% AR, 65%
Charitos et al. (2012)^20^	1760	44 ± 12	71	24/23/51	SC (44%), RR (56%)	35	N/A	7.1 ± 4.6	HR (AR vs AS), 2.3 (95% CI, 1.5-3.5); *P* <.001
Da Costa et al. (2014)^21^	414	31 ± 13	50	29/39/31	RR (86%), inclusion (14%)	7	External strip of Dacron/pericardium	8.2 ± 5.2	At 15 y: 91% overall
Martin et al. (2017)^25^	310	41 ± 11	78	73/19/7	RR (84%), inclusion (11%), SC (6%)	1	N/A	15.1 (IQR, 5.5-18.4)	HR (AR vs AS), 2.7 (95% CI, 1.4-5.1); *P* = .002
Ryan et al. (2011)^26^	160	42 ± 11	87	42/58/0	RR	38	Circumferential suture annuloplasty	AS, 4.5 ± 2.9; AR, 6.0 ± 3.2	At 10 y: AS, 95 ± 5%; AR, 67 ± 9%

*BAV*, Bicuspid aortic valve; *UAV*, unicuspid aortic valve; *QAV*, quadricuspid aortic valve; *AS*, aortic stenosis; *AR*, aortic regurgitation; *RR*, root replacement; *SC*, subcoronary; *N/A*, not available; *HR*, hazard ratio; *CI*, confidence interval; *IQR*, interquartile range.

∗ Percentage of patients in the cohort who presented with a bicuspid, unicuspid, or quadricuspid valve.

In the following sections, we examine the potential causes of the association between AR and premature autograft failure and ask whether they can be addressed at the time of surgery and in the early postoperative period. Furthermore, given that reoperation is only one of many important metrics to consider when evaluating the outcomes of valve surgery in young patients, we put these results in their broader context and examine the impact of preoperative AR on other critical endpoints, such as survival and quality of life.

### Mechanistic Insights

Although the association between preoperative AR and postoperative autograft dilatation has been clearly established, the underlying pathophysiology remains incompletely understood. It has been proposed—with little supportive evidence—that the presence of AR and a dilated aortic annulus may be a surrogate for genetic disease of the semilunar valves and great arterial walls, and that this genetic abnormality may impair adaptive remodeling of the pulmonary autograft, leading to early dilatation and failure. Indeed, following its implantation in the aortic position, the pulmonary autograft—which is a living structure—adapts and remodels in response to the drastic change in hemodynamic conditions compared with its native position within the pulmonary circulation. It has been suggested that a proportion of patients who present with AR and a dilated aortic annulus may have an unrecognized genetic vascular anomaly that impairs this process. This “genetic” hypothesis is supported by the observation in some series that surgical maneuvers aimed at stabilizing the aortic annulus in the hope of preventing autograft dilatation appear to be ineffective in these patients. Indeed, early on in their experience with the Ross procedure, David and colleagues^31^ recognized that the aortic and pulmonary roots often had a size mismatch in patients with AR, and that this was a cause of early autograft failure. Following this observation, the authors began to systematically adjust the size of the aortic annulus before autograft implantation whenever such a mismatch was present. This was achieved by way of subcommissural plication of the noncoronary sinus and partial annuloplasty with a Dacron strip. Importantly, this partial annuloplasty was performed along the fibrous portion of the left ventricular outflow tract. These maneuvers were effective in preventing early autograft dilatation and AR but did not prevent late autograft failure, leading the authors to conclude that AR and a dilated aortic annulus portend premature autograft failure that cannot be curtailed surgically.^24^

Several observations argue against this genetic hypothesis, however. First, that subcommissural plication did not prevent autograft dilatation should come as no surprise, as this technique has also proven ineffective in the context of aortic valve repair.^32^ This is because in AR, dilatation occurs mainly at the level of the muscular, rather than fibrous, portion of the annulus, an issue not addressed with subcommissural stitches or a Dacron strip along the aorto-mitral curtain. Second, none of the studies describing an association between preoperative AR and early autograft failure have reported the use of a strict postoperative blood pressure control regimen. Third, studies have shown that in patients who suffer postoperative autograft dilatation and failure, most of the dilatation is incurred by hospital discharge, suggesting that technical factors might be at play.^33^ Fourth, recent cardiac magnetic resonance imaging studies have shown that when using a patient-specific tailored approach (see below), the autografts of patients presenting with AR show similar dimensions, stiffness, and geometry at 1 year after the Ross procedure as seen in patients presenting with AS.^34^ These findings suggest that under the right conditions, the pulmonary root of patients with AR is capable of the same adaptive remodeling as that of patients with AS. Collectively, these 4 factors suggest that with proper adjustments to the surgical technique and postoperative management, excellent results can be achieved with the Ross procedure in patients with AR. In the next section, we review 3 strategies that have been proposed to improve the durability of the Ross operation in these patients.

### External Support of the Pulmonary Autograft

Several strategies have been proposed to mitigate the risk of early autograft dilatation and failure in patients with AR. Broadly speaking, these strategies consist in providing external support to the autograft and ensuring strict postoperative blood pressure control. The most promising and widely used approaches include autologous support of the autograft using the patient's own aortic root in the “inclusion” technique (Figure 1, *A*),^29^ external support of the autograft using a prosthetic Dacron tube (Figure 1, *B*),^35^ and a “tailored” surgical approach (Figure 1, *C*).^36^^,^^37^ Each of these approaches has advantages and limitations.

**Figure 1:**
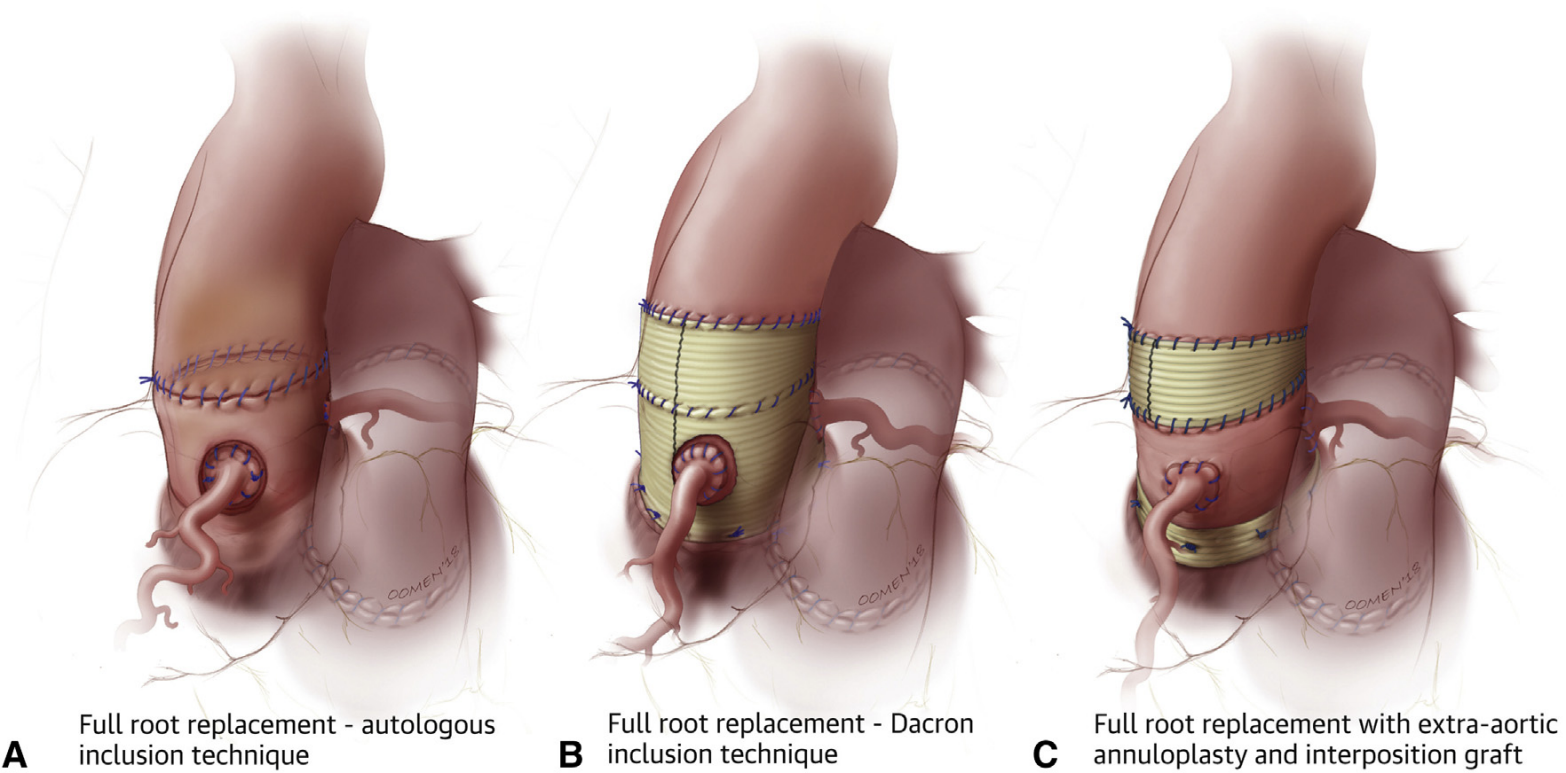
Technical modifications of the Ross procedure aimed at preventing late autograft dilatation and insufficiency. A, Autologous inclusion technique. B, Dacron inclusion technique. C, Tailored surgical approach.

### Autologous Support (Inclusion Technique)

In an effort to stabilize the neoaortic root and prevent autograft dilatation, several groups have proposed using the patient's own aortic root to provide external support to the pulmonary autograft (Figure 1, *A*). The largest study of this “autologous inclusion” technique comes from the Melbourne group, who reported their experience with this approach in 322 consecutive patients (median age, 40 years).^29^ The long-term results were impressive, with stable neoaortic root dimensions up to 15 years after surgery. Indeed, only 1.5% of patients had a maximum aortic root size >40 mm at follow-up, and none had an aortic root size >43 mm. Consistent with previous reports, patients who presented with preoperative AR and a dilated aortic annulus were at higher risk of increased neoaortic root diameters and reoperation at follow-up. Nonetheless, overall freedom from reoperation was 96% at 15 years, and none of the reinterventions were prompted by autograft dilatation.

In a subanalysis of this cohort focusing on 129 consecutive patients (mean age, 35 ± 11 years) who presented with BAV and AR, Skillington and colleagues^29^ reported a total of 11 reoperations (9 for recurrent AR, 2 for endocarditis), yielding overall freedom from reoperation of 85% at 20 years. In this analysis, most autograft failures occurred early (within the first 5 years after surgery). There was also a large era effect, with 45% of reoperations occurring in the first 25% of patients, before maturation of the authors' surgical strategies.

The Melbourne group's results with the autologous inclusion technique currently represent the best long-term outcomes of the Ross procedure in patients with AR, suggesting that when feasible, this approach is an excellent solution to stabilize the neoaortic root. The main limitation of this approach is that it is not well suited for patients with unicuspid or Sievers type 0 (180° commissures) bicuspid anatomy, especially when there is a large size discrepancy between the aortic and pulmonary roots.

### External Support With a Dacron Graft

To prevent autograft dilatation, several groups have proposed encasing the pulmonary autograft within a Dacron tube before implantation (Figure 1, *B*).^35^^,^^38^, ^39^, ^40^, ^41^ Both straight grafts and Valsalva grafts (Terumo Vascutek, Renfrewshire, United Kingdom) have been used in this context. This technique has shown good early results, with stable autograft dimensions up to 5 years.^29^^,^^38^, ^39^, ^40^, ^41^, ^42^ The main limitation of this approach is that it modifies the shape of the autograft root and impairs its natural dynamism, thereby potentially negating some of the main advantages of the Ross operation. Furthermore, studies have shown that the absence of mechanotransduction leads to extracellular matrix and smooth muscle cell disarray, which results in the autograft losing its elastic properties.^43^^,^^44^

Beyond these theoretical concerns, this approach also has a number of technical pitfalls. Distortion of the natural geometry of the autograft during its insertion in the Dacron tube can lead to early AR. Similarly, because the geometric height of the pulmonary cusps is longer than that of aortic cusps, one must ensure sufficient height of the commissures within the Dacron graft to avoid leaflet prolapse and early autograft failure.^45^ Another pitfall is the potential for the coronary arteries to be distorted or kinked by the Dacron graft. Yet another concern is the potential for blood to accumulate in the free space between the autograft wall and the Dacron graft, forming a hematoma that may compress the neoaortic root or create a nidus for infection. Finally, the use of Dacron has been associated with an increased inflammatory reaction around the pulmonary autograft, which can lead to early dysfunction and limit the long-term benefits of the Ross operation.^46^ The use of alternative synthetic materials, such as GoreTex, has been proposed, but long-term results are not available.^47^

In light of these limitations, we do not currently advocate the systematic use of this technique. Nevertheless, we see value in this approach, as it may open the possibility of extending indications for the Ross procedure to patients previously deemed to have an absolute contraindication, such as young patients with a connective tissue disorder and a nonsparable valve.^48^

### Tailored Surgical Approach

In an effort to minimize the risk of dilatation while maintaining autograft root dynamism—and hence optimal hemodynamics and left ventricular strain—we advocate the use of a bespoke approach by which surgical management is tailored to each individual patient. This strategy focuses on known risk factors for autograft dilatation and targets them according to the patient's individual anatomy and characteristics. This tailored surgical approach (Figure 1, *C*) has been described in detail elsewhere.^36^^,^^37^ In brief, before implantation, the autograft is trimmed, leaving the least possible amount of infundibular muscle. The autograft is implanted in a subannular position, so that it may be supported by the native aortic annulus. The autograft is oriented so that the left-facing sinus (ie, thinnest wall) sits in the left coronary sinus. Before implanting the autograft, it is critical to assess commissural height and symmetry. This is of particular importance in patients with bicuspid or unicuspid valves, who frequently display asymmetrically distributed raphes. Raphes are by definition lower in height than true commissures. When implanting the autograft, it is critical to ensure commissural height and angle symmetry, irrespective of the patient's native anatomy. Neocommissures should be created 120 degrees from one another. Failure to do so can result in distortion of the pulmonary autograft, a potential cause of AR or leaflet prolapse, resulting in early autograft failure. In patients with AR, we systematically perform an extra-aortic annuloplasty using a complete circular Dacron ring to further stabilize the aortic annulus.^49^ In addition, the ring annuloplasty readily corrects any significant (>2 mm) size mismatch between the aortic and pulmonary annuli, as the size of the ring is chosen based on the diameter of the pulmonary autograft annulus. This approach provides more robust annular stabilization compared with suture annuloplasty or partial rings. It also preserves the mobility of the autograft sinuses, in contrast to the wrapped Ross technique. To provide further support, we use the native noncoronary sinus and left–right commissure as a “loose external jacket.” In addition, to minimize the amount of pulmonary artery wall exposed to systemic pressures, we trim the pulmonary artery immediately above the commissures.

Given that sinotubular dilatation is a known mechanism of autograft failure, we advocate proactive management of the ascending aorta in patients with an ascending aortic diameter >36 mm, or a size mismatch of >3 mm to 4 mm between the ascending aorta and autograft sinotubular junction. This is achieved by interposing a short Dacron graft between the autograft and the ascending aorta, which stabilizes the sinotubular junction and prevents any late autograft dilatation in the event of native aortic dilatation.

In addition to these technical details, strict postoperative blood pressure control is another cornerstone of this tailored approach. We believe this is essential to allow the autograft to adapt to its new hemodynamic environment and avoid early dilatation during this adaptive phase. To this end, we have implemented a rigorous patient-centered remote blood pressure monitoring protocol, aiming for a systolic blood pressure ≤110 mm Hg for the first 12 months after surgery. Our experience has shown that a majority of patients who are discharged with adequate blood pressure control will require additional adjustments of antihypertensive medication dosages, particularly in the first month after surgery. Of note, these medication adjustments are more frequent in patients with AR, further highlighting the importance of continuous patient monitoring beyond the hospital stay in this higher risk population.

Since 2011, we have performed >500 Ross procedures using this approach, with excellent mid-term results and no sign of autograft dilatation. A recent analysis found no differences in autograft root dimensions between patients with preoperative AR versus AS up to 7 years after surgery, suggesting that these maneuvers are effective at preventing the early dilatation and failures observed in previous studies.^36^ Continued follow-up is warranted to confirm the long-term stability of autograft dimensions and determine the viability of this approach.

### Broader Perspective

As summarized in Table 1, several studies have drawn an association between preoperative AR and autograft failure, especially in the presence of a dilated aortic annulus. As such, patients with these anatomic characteristics represent a suboptimal substrate of candidates for the Ross procedure. However, when examining these data, a few important points should be kept in mind. First, the current evidence linking preoperative AR to autograft failure is derived from cohorts that antedate the technical refinements summarized in the previous section (Figure 2) and thus likely represents a “worst-case scenario.” Second, even in the context of this “worst-case scenario,” it is noteworthy that all large contemporary Ross series have included a significant proportion of patients with AR, ranging from 20% to 50%, and yet rates of reoperation have been rather low, ranging from 1% to 2% per patient-year.^12^ Third, and perhaps most important, a large body of evidence suggests that this burden of reoperation after the Ross procedure does not amputate long-term survival, and that despite higher rates of reoperation, the survival benefit observed after the Ross procedure is maintained in AR (Figure 2).^13^ This is demonstrated by the fact that the majority of contemporary cohort studies with long-term follow-up (≥15 years) have reported similar survival between patients who undergo the Ross procedure and the age- and sex-matched general population, despite including a significant proportion of patients with AR.^13^ Furthermore, in a large Ross cohort with a median follow-up of 15 years, Martin and colleagues^25^ reported equivalent long-term survival between patients who required Ross-related reintervention and those who did not. As such, reoperation should not be seen as an absolute failure in patients who undergo the Ross procedure. These patients are young adults and are likely to require multiple interventions in their lifetime, irrespective of the choice of initial procedure. Instead, the focus should be on restoring normal life expectancy and quality of life. The Ross procedure is the only aortic valve replacement operation that has been shown to restore normal life expectancy in young and middle-aged adults.^13^

**Figure 2:**
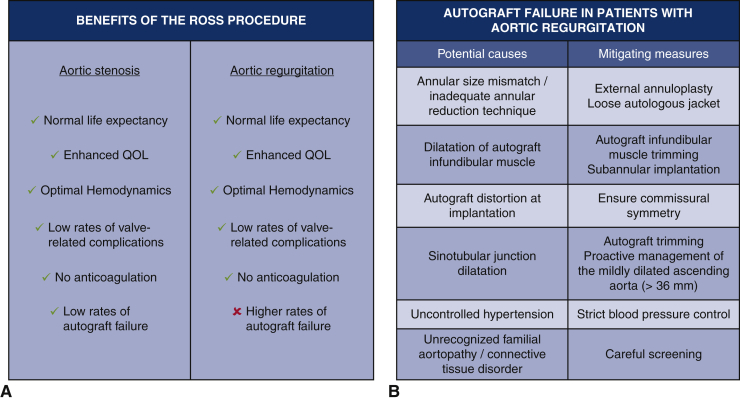
A, Benefits of the Ross procedure in patients with aortic stenosis and aortic regurgitation. B, Potential causes of autograft failure in patients with aortic regurgitation and mitigating measures. *QOL*, Quality of life.

## Conclusions

The Ross procedure is the best operation to treat AS in young and middle-aged adults^19^; however, its role in nonrepairable AR is more controversial. Several studies have demonstrated that patients with AR are at elevated risk of autograft dilatation and failure. Nevertheless, the survival benefit observed in AS—which is secondary to the unique biological and hemodynamic properties of the living autograft—is preserved in patients with AR. As a result, we believe the Ross procedure to be a better option than prosthetic AVR in selected patients with AR in terms of survival, quality of life, and hemodynamics. Importantly, we believe that the risk of autograft dilatation can be significantly mitigated with technical refinements and adjunctive measures as described in this review. Consequently, a tailored Ross procedure represents an excellent proposition in young patients with nonrepairable AR.

### Conflict of Interest Statement

The authors reported no conflicts of interest.

The *Journal* policy requires editors and reviewers to disclose conflicts of interest and to decline handling or reviewing manuscripts for which they may have a conflict of interest. The editors and reviewers of this article have no conflicts of interest.
